# Visual Attention and the Neuroimage Bias

**DOI:** 10.1371/journal.pone.0074449

**Published:** 2013-09-05

**Authors:** D. A. Baker, N. J. Schweitzer, Evan F. Risko, Jillian M. Ware

**Affiliations:** 1 School of Social and Behavioral Sciences, Arizona State University, Tempe, Arizona, United States of America; 2 Department of Psychology, University of Waterloo, Waterloo, Ontario, Canada; Hospital General Dr. Manuel Gea González, Mexico

## Abstract

Several highly-cited experiments have presented evidence suggesting that neuroimages may unduly bias laypeople’s judgments of scientific research. This finding has been especially worrisome to the legal community in which neuroimage techniques may be used to produce evidence of a person’s mental state. However, a more recent body of work that has looked directly at the independent impact of neuroimages on layperson decision-making (both in legal and more general arenas), and has failed to find evidence of bias. To help resolve these conflicting findings, this research uses eye tracking technology to provide a measure of attention to different visual representations of neuroscientific data. Finding an effect of neuroimages on the distribution of attention would provide a potential mechanism for the influence of neuroimages on higher-level decisions. In the present experiment, a sample of laypeople viewed a vignette that briefly described a court case in which the defendant’s actions might have been explained by a neurological defect. Accompanying these vignettes was either an MRI image of the defendant’s brain, or a bar graph depicting levels of brain activity–two competing visualizations that have been the focus of much of the previous research on the neuroimage bias. We found that, while laypeople differentially attended to neuroimagery relative to the bar graph, this did not translate into differential judgments in a way that would support the idea of a neuroimage bias.

## Introduction

Advances in neuroimaging technologies, such as MRI and functional MRI, have given scientists previously unimaginable access to how the brain functions. This privileged access, however, has brought with it a host of serious ethical concerns. Among these concerns is how the lay public understands neuroimaging information [1], [2], [3], [4], [5]. For example, scientists, legal scholars, and policy makers have expressed apprehension about providing neuroimages to lay persons as evidence of the credibility of specific knowledge, provided this particular visual aid might unduly bias beliefs in favor of the legitimacy of that particular claim [2], [6], [7], [8], [9], [10]. Research designed to examine this potential bias has found mixed results: a number of well-cited studies have suggested that this concern has merit [8], [11], [12], [13], while other, more recent findings suggest that neuroimages do not bias lay-judgments [14], [15], [16], [17], [18]. As neuroscience continues to offer new and more detailed visual evidence to support linkages between behavior and brain physiology (whether in a legal, scientific, or pop-culture setting), it becomes increasingly important to understand any incongruent persuasive impact these images may have.

The potential for neuroimages to unfairly bias a layperson’s judgments of scientific credibility has been the subject of a relatively large number of scientific studies. Three experiments conducted by McCabe and Castel [11] demonstrated such a biasing effect when they presented participants with short articles describing fictitious neuroscience studies and asked them to make judgments about the favorability of the articles. The findings suggested that participants judged articles accompanied by a neuroimage more favorably than those accompanied by the graphical representation. In apparent concert with McCabe and Castel’s findings, Weisberg, Keil, Goodstein, Rawson, and Gray [13] also found a potential biasing effect of neuroimages. In their study, students presented with descriptions of psychological phenomena were more satisfied with the descriptions when they were accompanied by neuroscientific information, including neuroimages, even when the accompanying information was irrelevant to the psychological phenomena itself.

Investigating how neuroimages may impact decision-making within a legal framework serves as an excellent context under which to study whether the persuasive weight given to a neuroimage exceeds the weight of the scientific information it actually conveys. For example, Gurley and Marcus [8] examined the persuasiveness of neuroimaging on mock jurors’ willingness to find a criminal defendant Not Guilty by Reason of Insanity (NGRI). When mock jurors were presented with evidence that included expert testimony that was neuroscience-based (a structural neuroimage plus an expert’s testimony), they were 1.3 times more likely to render a verdict of NGRI than the same expert testimony that lacked neuroscience-based evidence. However, while this study does speak to the persuasiveness of neuroscience-based testimony, the potentially independent persuasive effects of the neuroimages and neuroscientific language is difficult to disentangle. Greene and Cahill [15] investigated how brain scans would impact mock jurors when presented as evidence to support claims that brain abnormalities reduce a dangerous offender’s culpability. They found that the presence of neuroimages significantly reduced the likelihood that an individual would recommend a death sentence for the defendant. Furthermore, McCabe et al. [12] demonstrated the persuasiveness of neuroscience based evidence, even in the absence of a neuroimage. They presented mock jurors with a criminal trial scenario that manipulated evidence type to include fMRI, polygraph, or thermal facial information to support a lie detection claim. They found significantly more guilty verdicts were rendered in the fMRI information condition suggesting that neuroscience information (without an image) may have an independent biasing effect, though this effect was negated by a statement that questioned the validity of fMRI techniques.

While the aforementioned studies show a persuasive effect of neuroimages (or neuroscience based information), another body of work that has looked directly at the independent impact of neuroimages on layperson decision-making has shown evidence to the contrary. Schweitzer, et al. [18] conducted a series of experiments in which mock jurors evaluated expert testimony. In this series of experiments, the researchers manipulated the type of expert testimony, image type, image “glitziness,” seriousness of the crime, and the amount of non-expert testimony presented. Consistent with previous research, they did find a significant impact of neuroscience-based evidence, but in all four experiments, they failed to find an individual biasing effect of neuroimages. Schweitzer and Saks [17] also failed to find a neuroimage bias across individuals evaluating a written mock trial. Interestingly, although no significant differences in judgments were found between individuals who saw the neuroimage and those who did not, those who did not reported that the most helpful type of evidence would have been neuroimagery (more so than DNA evidence, eyewitness testimony, family background and work history), suggesting that, despite the lack of impact on decision making, individuals do consider neuroimagery to possess helpful information. Moving outside a legal context, Gruber and Dickerson [19] reexamined the persuasiveness of a fake scientific news article (using similar methodology to McCabe and Castel [11]) when it was accompanied by an fMRI and found it to be no more or less persuasive than when the article was accompanied by other image types or no image at all. While the existence of a biasing influence of neuroimages (versus non-neuroimage representations of the same information) on high-level decisions is unclear, in the present investigation we set out to assess the influence of neuroimages on the distribution of attention in the context of a legal decision. In other words, do individuals attend to neuroimages differently than non-neuroimage representations of the same information? The idea that the presentation of neuroimages might bias attention has indirect support in research on eye movements (i.e., a measure of overt attention). For example, Berlyne [20] demonstrated that individuals look longer at novel, complex images. Indeed, novelty is often used to attract individuals’ attention in marketing contexts [21], [22], [23], [24]. Images of the brain, of course, represent just such a stimulus for many potential jurors.

Finding an effect of neuroimages on the distribution of attention would provide a potential mechanism for the influence of neuroimages on higher-level decisions (when present). For example, if neuroimages “capture” individuals’ attention, this could influence subsequent decisions by biasing memory for the information encompassed in the image. There exists a voluminous literature attesting to the association between attention and memory. For example, when attention is divided between encoding a list of words and a secondary task, a marked decrease in recall is observed relative to a focused attention condition [25], [26]. In addition, when presented with an array of objects, the amount of attention paid to a given object predicts later recall of that object [27]. Thus, a novel, salient visual stimulus could attract/hold attention, and attention can influence the likelihood of later recall. Returning to the legal context, if a neuroimage is more likely to capture/hold attention, then the information in that image may be more likely to be recalled at a point in time when a decision (e.g., guilt) needs to be made. Alternatively, failure to find an attentional bias toward neuroimages would suggest that any influence of neuroimages on higher-level decisions would be due to differences in how the stimulus was attended to.

### The Present Experiment

The aim of the present study is to investigate whether participants visually attend to neuroimages in a different manner than to a bar graph conveying similar information, and whether patterns of inspection influence later decisions. Eye-tracking was used to record the duration and number of fixations participants made to different information sources in the context of reading a short vignette (describing a defendant’s crime and his diagnoses of mental illness) that included either a structural neuroimage or a bar graph that presents similar information. Eye-tracking is often used as a moment-by-moment index of what an individual is attending to in a visual display (for a review see Rayner [28]) and as such has the potential to provide a sensitive test of any differences in attention paid to neuroimages versus a more mundane bar graph. As such, in the present investigation we explore three questions (a) do individuals attend differently to neuroimages relative to a bar graph that presents similar information (b) can neuroimages bias decisions in a legal context and finally (c) is there any relation between how individual’s attend to neuroimages (or bar graphs) and their decisions in a legal context. It is important to note that the issue of an attentional bias to neuroimages (see question (a) above) and the relation between attention and subsequent decisions (see question (c) above) are independent. That is, there might be a bias to attend to neuroimages but no influence of attention on subsequent decisions, or there might be no bias to attend to neuroimages but a relation between attention and subsequent decisions.

## Methods

### Participants

The research was determined to be exempt by the Institutional Review Board of Arizona State University, as no foreseeable risk to the participants was present, and personally identifying information was not collected. The IRB of Arizona State University does not require that written consent be obtained from participants when the research has been granted exempt status. As such, prior to volunteering for the study, participants were asked to read a consent letter in which the study was described and the voluntary nature of participation was made explicit. Seventy-three undergraduate students from Arizona State University were recruited to participate in the study for either class credit or $5.00. The sample was 76.7% female and had a mean age of 22.94 years.

### Design and Materials

**Figure 1:**
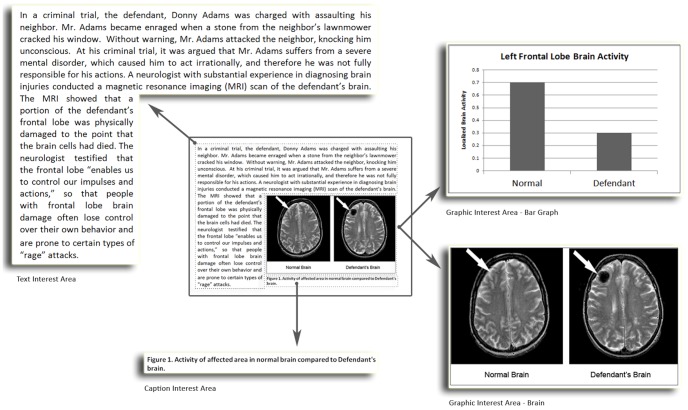
Trial Scenario With Interest Areas. Participants were presented with a brief written summary that described the case of “Donny Adams.” Based on random assignment, either the neuroimage or bar graph was presented with the summary. Approximation of individual interest areas used to record eye movement data areas have been enlarged for illustrative purposes.

The stimulus material consisted of a brief summary that described the case of “Donny Adams”, who was charged with assaulting his neighbor after a stone from the neighbor’s lawn mower cracked Mr. Adams’ window. During the trial, the defense presented evidence that Mr. Adams suffers from a mental disorder, which causes him to act irrationally, and limits his control over his own behavior. Expert testimony from an experienced neurologist was introduced in which the results of a magnetic resonance imaging (MRI) scan revealed significant damage to the frontal lobe of Mr. Adams’ brain. The neurologist explained that such damage could impair a person’s ability to control their impulses and actions, suggesting that the damage to Mr. Adams’ brain may have caused the defendant to lose control over his actions. Embedded within the text was either a black and white graph comparing the “localized brain activity” of a normal brain to the that of the defendant’s brain (which indicated significantly lower activity levels) or a black and white MRI scan of a normal brain positioned next to the defendants brain (which had a significantly sized dark area not present in the normal brain). These stimuli are included as Figure 1. For eye-tracker recording purposes individual interest areas were then defined around the text and the image area such that the number of fixations and the total dwell time for each interest area could be captured separately (see Figure 1).

### Apparatus and Procedure

The trial scenarios were presented on a 17″ color monitor connected an SR Research Eyelink 1000 eye-tracker. The camera was positioned in front of the monitor facing the participant and a headrest was used to keep participants still during the recording session. Participants were run individually and calibrations of the eye-tracking settings were conducted for each participant prior to beginning the trial. Participants were randomly assigned to either the MRI or graph condition. Participants read brief instructions indicating they would be reading a summary of a criminal case and then answering questions about what they had read; the following screen presented the trial scenario and image. Eye-tracking data were collected only while the participant was attending to either the text or image. Upon completion of the reading task, participants were asked to render a guilty/not guilty (1) verdict; to make judgments (using a 7-point Likert scale) about the extent to which they felt the defendant should be (2) punished for his crime and would be likely to (3) recidivate if left untreated. Finally, participants were asked to fill out a brief demographic survey.

## Results

### Eye Movements

**Figure 2:**
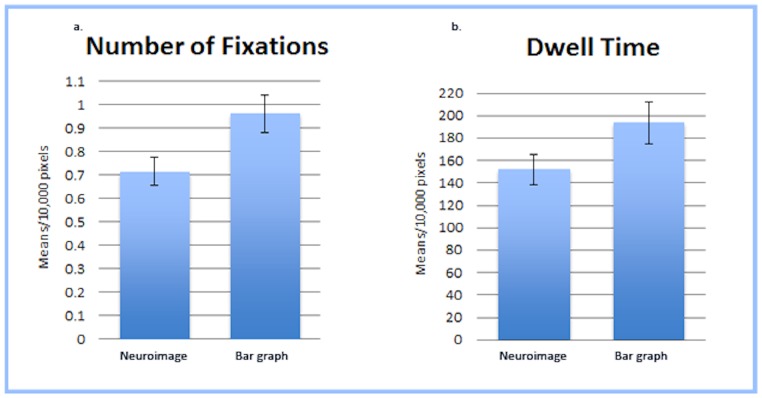
Fixation Count and Dwell Time for Image Interest Area. (a) Mean Number of Fixations (per 10,000 pixels) on the Scenario’s Image Interest Area. Participants in the bar graph condition fixated on the image significantly more often than those in the neuroimage condition. (b) Mean Dwell Time (per 10,000 pixels) on the Scenario’s Image Interest Area. Participants in the bar graph condition tended to dwell longer on the image than participants in the neuroimage condition, however this result was not significant.

Before beginning statistical analyses on the eye-tracking data, we standardized all measures to “per 10,000 pixels” (noted as 10 kpx) to account for small differences in the pixel count between the defined interest areas. To determine if individuals attend differently to neuroimages relative to a bar graph that presents similar information, we first conducted a between-subjects analysis of variance comparing Number of Fixations (NF) on the scenario’s image interest area across image type. (Note: An analysis of variance was also conducted examining Number of Fixations (NF) and Dwell Time (DT) on the text of the scenario between participants who saw the graph and those who saw the neuroimage and no significant differences were found.) Results indicated that participants in the graph condition fixated on the image more often (*M* = .964 per 10 kpx; as mentioned above means are reported per 10,000 pixels) than those in the neuroimage condition (M = .716 per 10 kpx; *F*(1,70) = 6.114, *p* = .016, *n_p_^2^* = .080; see Figure 2a). (Note: This analysis was also conducted using the NF recorded for the reading portion of the scenario as a covariate in order to control for the total amount of time participants spent on the entire page, but this did not substantively change the results, *F*(1,69) = 6.847, *p* = .011, *n_p_^2^* = .090.) This analysis was then repeated for Dwell Time (DT) and we found that although participants in the graph condition tended to dwell longer on the image (M = 193.57 ms per 10 kpx) than participants in the neuroimage condition (*M* = 151.78 ms per 10 kpx), this result was not significant *F*(1,70) = 3.367, *p* = .071, *n_p_^2^* = .046; see Figure 2b). (Note: As with the previous test, this analysis was also conducted using the DT recorded for the reading portion of the scenario as a covariate and this did not substantively change the results, *F*(1,69) = 3.276, *p* = .075, *n_p_^2^* = .045.).

**Figure 3:**
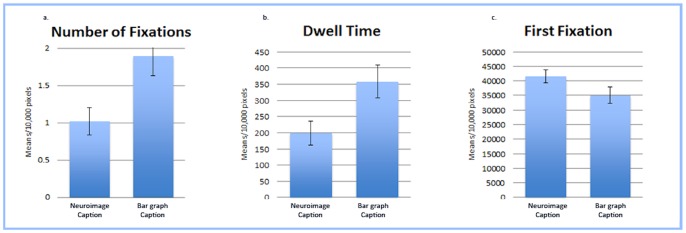
Fixation Count and Dwell Time for Caption Interest Area. (a) Mean Number of Fixations (per 10,000 pixels) on the Caption Interest Area. Participants fixated on the image caption significantly more often when it was paired with the bar graph than when it was paired with the neuroimage. (b) Mean Dwell Time (per 10,000 pixels) on the Caption Interest Area. Participants dwelled on the image caption significantly longer when it was paired with the bar graph than when it was paired with the neuroimage. (c) Mean Time (per 10,000 pixels) until First Fixation on the Caption Interest Area. The amount of time participants took to initially direct their gaze on the caption was not significantly different between the bar graph and neuroimage.

**Figure 4:**
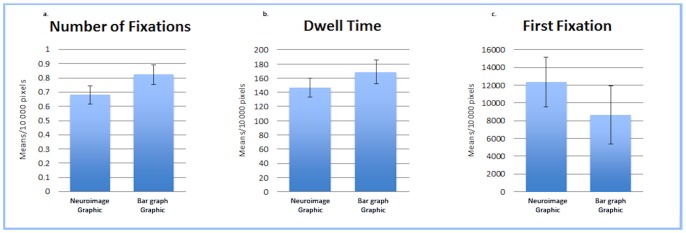
Fixation Count and Dwell Time data for Graphic Interest Area. (a) Mean Number of Fixations (per 10,000 pixels) on the Graphic Interest Area. The number of times participants fixated on the graphic area did not significantly differ between the bar graph and neuroimage conditions. (b) Mean Dwell Time (per 10,000 pixels) on the Graphic Interest Area. The amount of time participants dwelled on the graphic area did not significantly differ between the bar graph and neuroimage conditions. (c) Mean Time (per 10,000 pixels) until First Fixation on the Graphic Interest Area. The amount of time participants took to initially direct their gaze on the graphic area was not significantly different between the bar graph and neuroimage.

To further explore the influence of image type on eye movements, we divided the eye-movement data for our images into two separate interest areas: one encompassing the image caption and one encompassing the graphic (see Figure 1). We then conducted a set of one-way analyses of variance comparing NF and DT on the individual caption and graphic interest areas. The NF on the captions varied significantly by image type with individuals fixating on the graph caption significantly more often (*M* = 1.89 per 10 kpx) than the neuroimage caption (*M* = 1.02 per 10 kpx), *F*(1,70) = 7.31, *p* = .009, *n_p_^2^* = .095 (see Figure 3a). The NF on the graphic area, however, did not significantly differ by image type (*p* = .132; see Figure 4a). The DT on the captions also varied significantly by image type with individuals fixating on the graph caption significantly longer (*M* = 358.61 ms per 10 kpx) than the neuroimage caption (*M* = 198.66 ms per 10 kpx), *F*(1,70) = 6.47, *p* = .013, *n_p_^2^* = .085 (see Figure 3b). However, once again, the DT on the graphic area did not significantly differ by image type (*p* = .319; see Figure 4b).

In addition to NF and DT we also conducted an analysis of first fixation time (FF; the time it took for the participant’s gaze to initially enter an interest area) between the bar graph and neuroimage. Because the previous analysis demonstrated differences between the graphic and caption interest areas, to investigate differences in FF time we once again analyzed the graphic and caption interest areas separately. No difference in the FF was found between the graph (*M* = 35071.31 ms) and the neuroimage captions (*M* = 41647.12 ms; *p* = .078; see Figure 3c), nor the graph (*M* = 12337.08 ms) and neuroimage graphics (*M* = 8640.89 ms; *p* = .395; see Figure 4c). This indicates that the type of image (graph or neuroimage) did not differentially impact the amount of time individuals used to direct their gaze toward the graphic or caption areas.

### Influence of Image Type on Verdict, Punishment, and Recidivism Decisions

**Figure 5:**
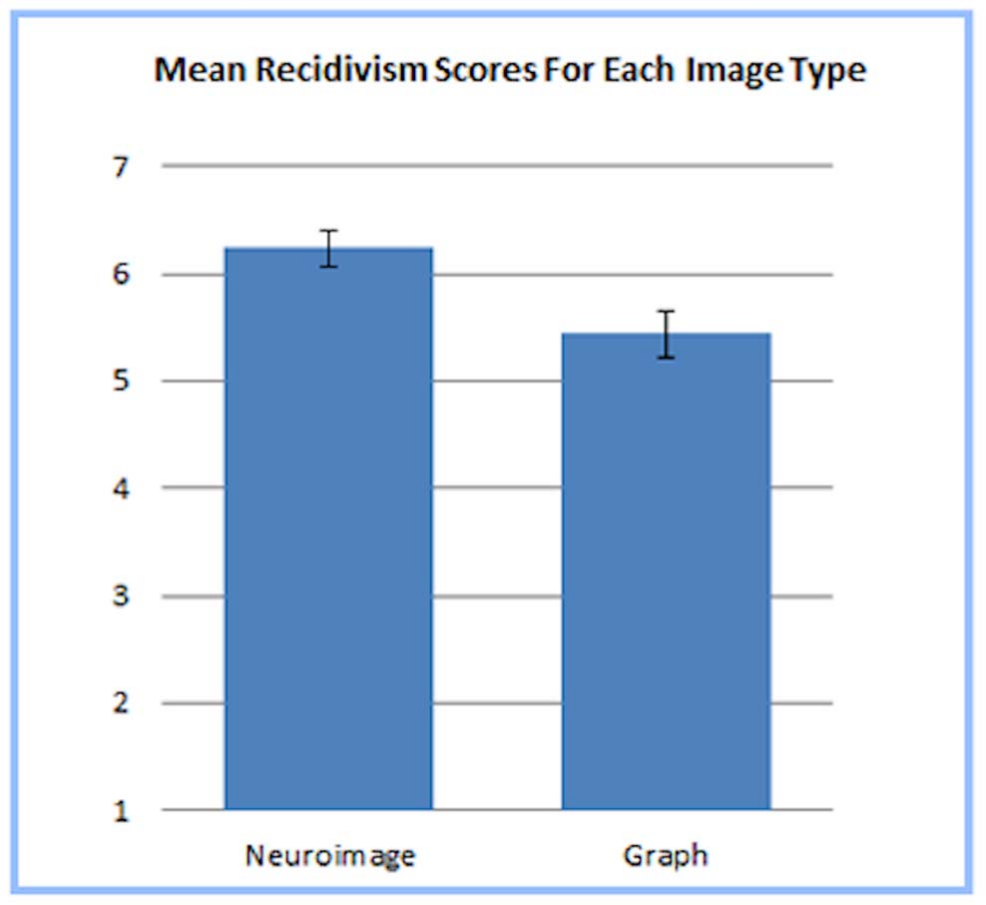
Mean Recidivism Scores For Each Image Type. Participants who viewed the neuroimage reported a stronger belief that the defendant in the scenario was likely to recidivate than participants who viewed the bar graph.

To examine if our neuroimages biased decisions within the legal context of our scenario, a logistic regression was conducted using Image Type to predict Verdict and indicated that the (1) Verdicts rendered by participants did not differ as a function of image type, *B* = .405, *p* = .438. Individual analyses of variance compared participant responses on the questions regarding (2) Punishment and (3) Recidivism and found that although Punishment was not impacted by Image Type, Recidivism did significantly differ between neuroimage and graph conditions, *F*(1,71) = 8.461, *p* = .005, *n_p_^2^* = .106. In other words, the neuroimage did not have a differential impact on whether participants felt the defendant should be found guilty or how severely participants felt the defendant should be punished; however, participants who saw the neuroimage viewed the defendant as more to likely to recidivate (*M* = 6.24) than those who saw the graph (*M* = 5.44; see Figure 5).

### Relation between Eye Movements and Verdict, Punishment, and Recidivism Decisions

In the final stage of our analyses, we investigated whether eye movements during inspection of the scenarios could predict any of our three dependent variables: (1) Verdict, (2) Punishment, and (3) Recidivism. We first conducted a binary logistic regressions using NF, DT, and FF (for the separate graphic and caption interest areas) as predictors of (1) Verdict and found that they had no impact on verdict decisions (all *p*s>.096). Second, we conducted a linear regression of NF, DT, and FF on judgments of (2) Punishment and on judgments of (3) Recidivism and this also yielded non-significant findings (all *p*s >.299).

Although the eye-movement data did not directly predict any of the three dependent variables, we focused on the influence of Image Type on Recidivism judgments (where a significant effect of Image was found) and explored eye-movements for their potential as mediating link in this relation. Using a bootstrapped mediation analysis (the most powerful test of mediation [29]; for an overview, see [30]), the six visual attention variables (NF caption area, DT caption area, FF caption area; NF graphic area, DT graphic area, and FF graphic area) were entered as potential mediators between Image Type and Recidivism. If the relation between Image Type and Recidivism were due to attentional differences, then this would be indicated by a significant “indirect effect” through one or more of the measures of attention. However, none of these indirect effects proved to be significant, suggesting that that the link between Image Type and Recidivism is independent of the attentional differences that were present. See Table 1 for detailed results.

**Table 1:** Tests of the indirect effect of Image Type on Recidivism through various measures of visual attention.

Potential Mediator	Indirect Effect	Bootstrapped 95% CI	Standard p-value
NF Graphic	ab = −.094	[−.545 ….224]	*p = *.62
NF Caption	ab = .335	[−.386 … 1.232]	*p = *.42
FF Graphic	ab = −.002	[−.110 ….058]	*p = *.96
FF Caption	ab = .011	[−.138 ….184]	*p = *.88
DT Graphic	ab = .128	[−.063 ….606]	*p = *.45
DT Caption	ab = −.160	[−1.02 ….489]	*p = *.67

## Discussion

The present investigation has revealed a number of interesting results. We did not find an independent biasing effect of neuroimages on participants’ likelihood of rendering guilty verdicts nor on judgments about the degree to which a defendant should be punished. This is consistent with recent research demonstrating the non-persuasiveness of neuroimages [14], [16], [17], [18]. However, judgments of recidivism were influenced by the presence of a neuroimage. Specifically, participants who read the scenario paired with a neuroimage judged that the individual would be more likely to recidivate than did those who read the same scenario when it was paired with a graph. With respect to eye movements, individuals attended to the neuroimage and bar graph differently, but these differences were not what were predicted: although an overall attentional difference appeared to exist between the neuroimage and the bar graph, a closer look revealed that this difference was more specifically related to differences in attention to the image caption. In other words, individuals looked at the neuroimage and graph the same number of times and for the same amount of time; however, they looked at the captions significantly less frequently and for a shorter duration when it was paired with the neuroimage than when that same caption was paired with the bar graph. Finally, there was no evidence to suggest that the observed attentional differences between the neuroimage and bar graph influenced judgments or decisions. This was true in the case of recidivism (where we did find that the image type influenced judgments), and was also true for both guilt and punishment (where image type had no influence).

### Neuroimage Effect on Recidivism Judgments

The influence of neuroimages on recidivism judgments can potentially be attributed to differences between the types of evidence that might be considered when making judgments related to what a person has done in the past versus what a person might do in the future. Specifically, the presence of a neuroimage (relative to a graph) did not impact decisions directly related to the defendant’s past criminal behavior (i.e., was he guilty of having committed the crime, and should he be punished for having committed that crime) but did impact a decision related to the defendant’s future behavior. The latter judgment could prompt an individual to consider enduring qualities, such as a chronic psychiatric conditions or permanent brain damage, which might be viewed as providing clues about the defendant’s future behavior. Neuroevidence that describes the existence of a physical brain abnormality could act as such a source of information. Thus, the need to make a judgment about recidivism likely increased the relevance of the neuroevidence. As such, the recidivism judgment might have provided a more sensitive test of the potential biasing effect of the neuroimage. When considering neuroevidence represented by either a graph or neuroimage, it is likely that the anatomical nature of a neuroimage would more readily prime a biological mindset. By presenting individuals with an image that appears to depict an enduring physical characteristic of the individual’s body, this biological priming may strengthen the connection between ‘bad behavior of the past’ and ‘bad behavior in the future.’ This would explain the tendency for individuals who saw the neuroimage to report a stronger belief that the defendant was likely to recidivate.

### Attention Capture for Neuroimages

In reading the vignettes, individuals paid more attention (as indexed by eye movements) to the image’s caption when it was paired with the bar graph than when it was paired with the neuroimage; however, attention to the actual graphic depicting either a neuroimage or a bar graph did not differ. A number of potential hypotheses can be forwarded to explain the observed patterns. With respect to the neuroimage itself (without the caption), individuals may not pay greater attention to the neuroimage, as might be expected, because (1) neuroimages may not, as suggested earlier, be perceived as “novel” to participants, (2) both the neuroimage and the bar graph are perceived as equivalently complex and are therefore ignored or (3) the neuroimage and bar graph are perceived to be equivalently transparent such that all relevant information can be obtained with the same amount of attention. With respect to novelty, it is quite plausible that neuroimages of the type we have used here are not perceived as novel; information about neuroscience and neuroimages can be easily accessed on the internet and neuroimages are now featured on medical shows, pharmaceutical commercials, print ads, and on some products. Using Lexis-Nexis a search of news articles containing the phrase “fMRI” yielded 447 results for 2007 and 1284 news articles in 2012, a very large increase, suggesting that neuroimagery is rapidly entering the mainstream. Indeed, the initial research demonstrating the “seductive allure” of neuroimages was conducted in 2006–2008 whereas research that has failed to find such evidence has been more recent [14], [15], [16], [17], [18]. This pattern of results might suggest that the “seductive allure” has worn off with the decrease in novelty of neuroimages. With respect to complexity, the black and white neuroimage used in the study was designed to reflect a clear and simple difference between a normal and abnormal brain, as was the bar graphs, however, it is possible that laypersons feel generally unqualified to interpret neuroscientific information and therefore generally ignore the images themselves. Finally, with respect to transparency, considering primary versus secondary representations of data may shed light not only on why individuals attended to the neuroimage and bar graph equivalently, but also on understanding why individuals attended to the caption less when it was accompanied by the neuroimage. The neuroimage is arguably a primary source (more or less a picture of the raw data) of evidence about the brain defect; it concisely conveyed a message that required little effort to perceive (i.e., a big black hole in the brain is not normal); whereas the graph provided a secondary interpretation of the same data that required the individual to identify the meaning of the axes and bars, and to infer the conclusion being illustrated. Individuals looked more often and spent more time reading the caption when it was accompanied by the graph, indeed suggesting that the information conveyed by the bar graph alone was either not sufficient enough to interpret its meaning or was not needed because the information was present in the caption. On the other hand, individuals paid less attention to the caption when it was paired with the neuroimage, suggesting the neuroimage conveyed a message that could be perceived with the same amount of attention as the bar graph, but did not require the additional information conveyed in the caption. This could be interpreted to mean that, within the limited scope of information that was relevant to the scenario, the neuroimage was more “self-explanatory” than the bar graph. This unique finding does not suggest that the neuroimage was more meaningful, but rather that the simple message conveyed by the image (i.e. that the defendant has an abnormal brain) required less attentional resources to perceive when presented as a neuroimage rather than a bar graph.

### Conclusions and Future Directions

Our findings generally support recent studies that neuroimages are not significantly more compelling than other image types representing the same information, particularly when using neurological information to judge a person’s behavior. We also demonstrate that although individuals attend to neuroimages and graphs differently, these differences do not affect subsequent judgments or decisions. We did find, however, that neuroimages had an impact on judgments about a person’s future behavior, however it is unclear if this impact can be attributed to unwarranted credibility (i.e. “seductive allure”) as described by Weisberg et al. [13]. Rather, a number of other potential explanations should also be explored through future research. For example, the anatomical nature of neuroimagery may prime biologically deterministic thinking more so than a graph, a frame of mind that is likely to make connections between an enduring quality and predictions of future behavior. Or, when seeking information to extrapolate from, the concept of “abnormality” that is represented by a neuroimage may be easier to recall than when it is represented by a graph. Further research might also explore visual fixation patterns when individuals are given multiple visualizations of neurological data, as current research suggests that biasing neuroimage effects might be present only when individuals have competing points of comparison [31]. But, at present, our findings should further assuage fears that the lay public sees neuroimagery as an irresistible persuasive force, and that, at least in the visual sense, neuroimagery’s seductive powers may have faded.
